# Detection and enumeration of circulating tumor cells based on their invasive property

**DOI:** 10.18632/oncotarget.4454

**Published:** 2015-07-11

**Authors:** Haizhen Wang, Yannis Hara, Xingtong Liu, James M. Reuben, Yongzhuang Xie, Huaxi Xu, Guojun Bu, Yihua Pei, Vineet Gupta, Xiangwei Wu

**Affiliations:** ^1^ Department of Clinical Cancer Prevention, The University of Texas MD Anderson Cancer Center, Houston, Texas, USA; ^2^ Department of Hematopathology, The University of Texas MD Anderson Cancer Center, Houston, Texas, USA; ^3^ Fujian Provincial Key Laboratory of Neurodegenerative Disease and Aging Research, Institute of Neuroscience, College of Medicine, Xiamen University, Xiamen, Fujian, China; ^4^ Central Laboratory, Zhongshan Hospital, Xiamen University, Fujian, China

**Keywords:** circulating tumor cells, cell invasion assay, metastasis, functional property

## Abstract

Circulating tumor cells (CTCs) are in limited numbers and heterogeneous, making their detection, isolation, and enumeration a major challenge. To overcome these difficulties, we developed a novel method to detect and enumerate CTCs with invasive property. Our assay consists of three simple steps: enrichment, Matrigel invasion assay, and immunostaining. We have validated this method using mouse xenograft tumor models and confirmed its utility in human cancer patients. Our method does not require special equipment and antigen expression for CTC selection, is less likely to be affected by the heterogeneity of the CTCs, and could be applicable to virtually all cancers. Most important, our method enumerates invasive CTCs, which may allow more accurate correlations with clinical outcome and treatment response compared with other CTC detection methods.

## INTRODUCTION

CTCs are emerging as an important biomarker for monitoring cancer treatment response and predicting clinical outcome. CTCs are thought to contain cells capable of initiating metastatic tumor growth [1, 2], which is the cause of most cancer-related deaths [3]. The formation of metastasis from a primary tumor involves multiple processes, including the local invasion and migration of tumor cells and their entrance into the lymphatic system or blood circulation (intravasation), survival in circulation, extravasation into distant organs, and development into metastasis [1, 4, 5]. Analysis of CTCs may also facilitate our understanding of tumor invasion and metastasis. CTCs are also emerging as an important biomarker for personalized therapy. The potential applications of CTC enumeration include predicting the clinical outcome of cancer patients, detecting potential metastasis early, and monitoring treatment response [6]. However, the number of CTCs in the blood of cancer patients is very low — from 1 to 10s per milliliter among the billions of red blood cells and millions of leukocytes — so the detection, isolation, and analysis of these cells pose real challenges [7, 8].

The most common methods for enumerating CTCs are based on the expression of epithelial cell markers (such as epithelial cell adhesion molecule; EpCAM) on cancer cells and antibody-based capture and selection [1, 9]. One of these methods, Veridex’s CellSearch system, was cleared by the U.S. Food and Drug Administration for monitoring the effectiveness of treatment for patients with metastatic breast, colorectal, or prostate cancer [10–12]. Another method, ScreenCell, uses a filtration-based device that isolates CTCs from human whole blood based on cell size [13, 14]. However, CTCs are a heterogeneous population of cells and some undergo epithelial-to-mesenchymal transition, causing variation in the expression of cell markers and cell size [15]. Therefore, these methods cannot detect all CTCs. More important, some CTCs may be derived from mechanical shedding rather than active invasion, and some CTCs may lose their viability or functionality and thus become irrelevant to cancer metastasis. Therefore, the CTC counts generated by these methods may not be accurate in cancer outcome and treatment response.

A functional CTC separation method, called collagen adhesion matrix assay, can detect and isolate CTCs with an invasive phenotype [16–18]. This method is based on tumor cells’ ability to attach and ingest collagen adhesion matrix. No actual migration or invasion is involved in the assay. Therefore, the ability of these cells to invade is unknown.

Since functional CTCs enter circulation likely through migration and invasion, and maintain these properties for extravasation, we hypothesize that functional CTCs can be detected *in vitro* using an invasion assay. In this report, we describe the development and validation of a simple and cell surface marker-independent assay for detecting, enriching and enumerating invasive CTCs.

## RESULTS

### A Simple matrigel invasion assay detects CTCs in xenograft tumor mice

**Figure 1:**
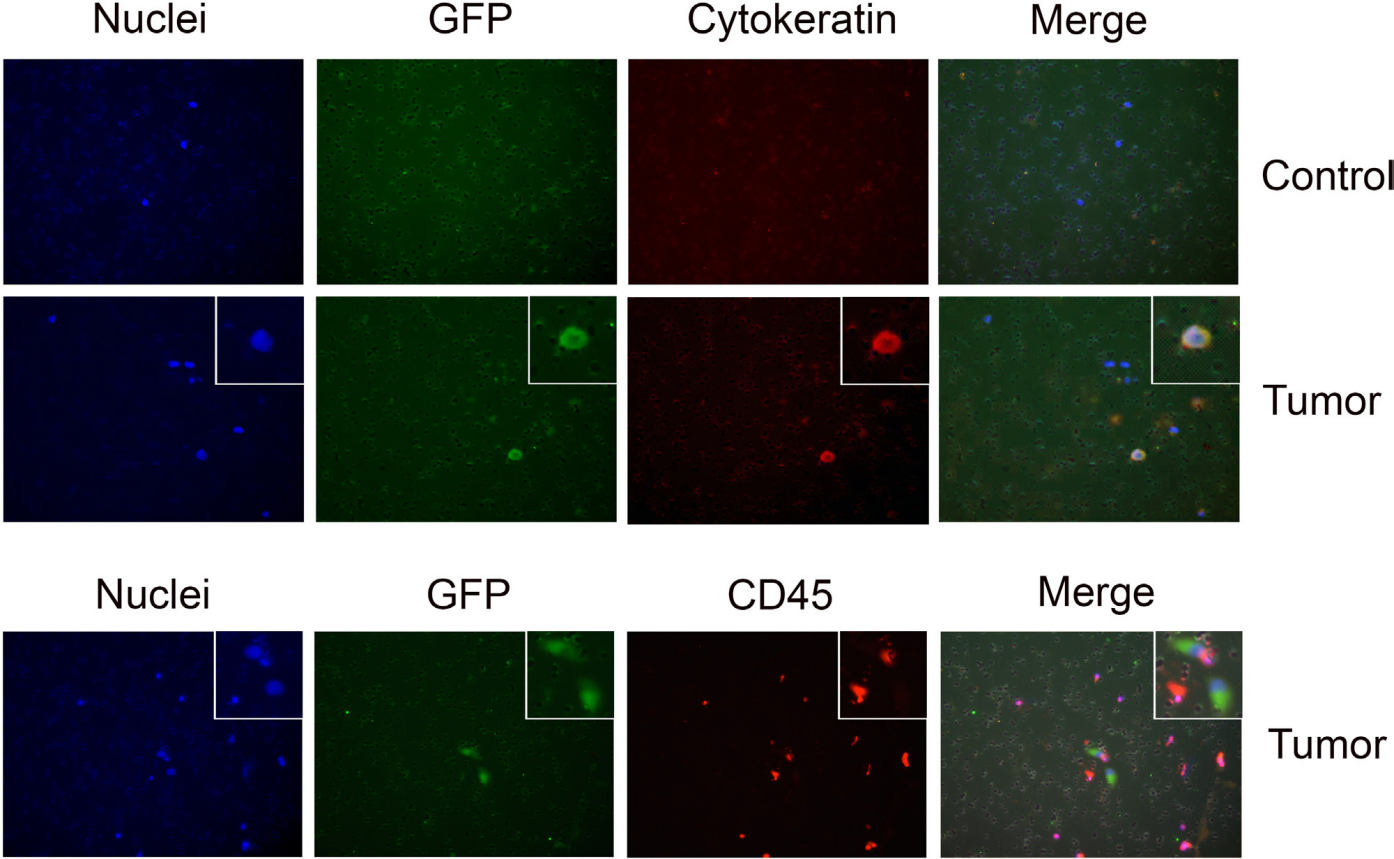
Detection of CTCs by InCTC. Mononuclear cells were enriched from blood samples of either a control nude mouse or a mouse with a xenograft tumor derived from NCI-H460-GFP cells were subjected to Matrigel invasion assay Nuclei were visualized by DAPI staining (blue). GFP-positive cells (green), cytokeratin-positive cells (red) and CD45-positive cells (red) were visualized under a fluorescent microscope. The experiments were repeated multiple times; representative pictures are shown.

To develop an assay for CTC isolation and detection that is based on these cells’ invasive property, we first established a murine model of metastatic tumor growth. The NCI-H460 non-small cell lung carcinoma cell line stably expressing enhanced green fluorescent protein (GFP) was used to generate flank tumors in nude mice. When a flank tumor reached 1 to 1.5 cm in diameter, it was surgically resected. Lung metastasis developed in 100% of the mice 3–4 months after resection (data not shown). Whole blood was collected at 4 months after the resection. The red blood cells were removed by density-gradient centrifugation, and the mononuclear cell fraction was subjected to a transwell invasion assay. Invasive cells were visualized under a fluorescent microscope, and the number of GFP-positive cells was counted (Figure 1). Although a few mononuclear cells were detected after the invasion assay, mice that had not undergone tumor cell injection (control mice) did not exhibit any GFP-positive cells, whereas GFP-positive cells were detected in the blood from tumor-bearing mice. To confirm that these GFP-positive cells were epithelial cells, we stained the invaded cells with anti-cytokeratin antibody and found that the all GFP-positive cells were also cytokeratin positive and CD45 negative, supporting the notion that they were tumor cells. Furthermore, all cytokeratin positive cells in the tumor-bearing mice were positive for GFP and no cytokeratin-positive cells were detected in control mice (Figure 1). We named this invasion-based CTC discovery method “InCTC”.

### Characterization of InCTC

**Figure 2:**
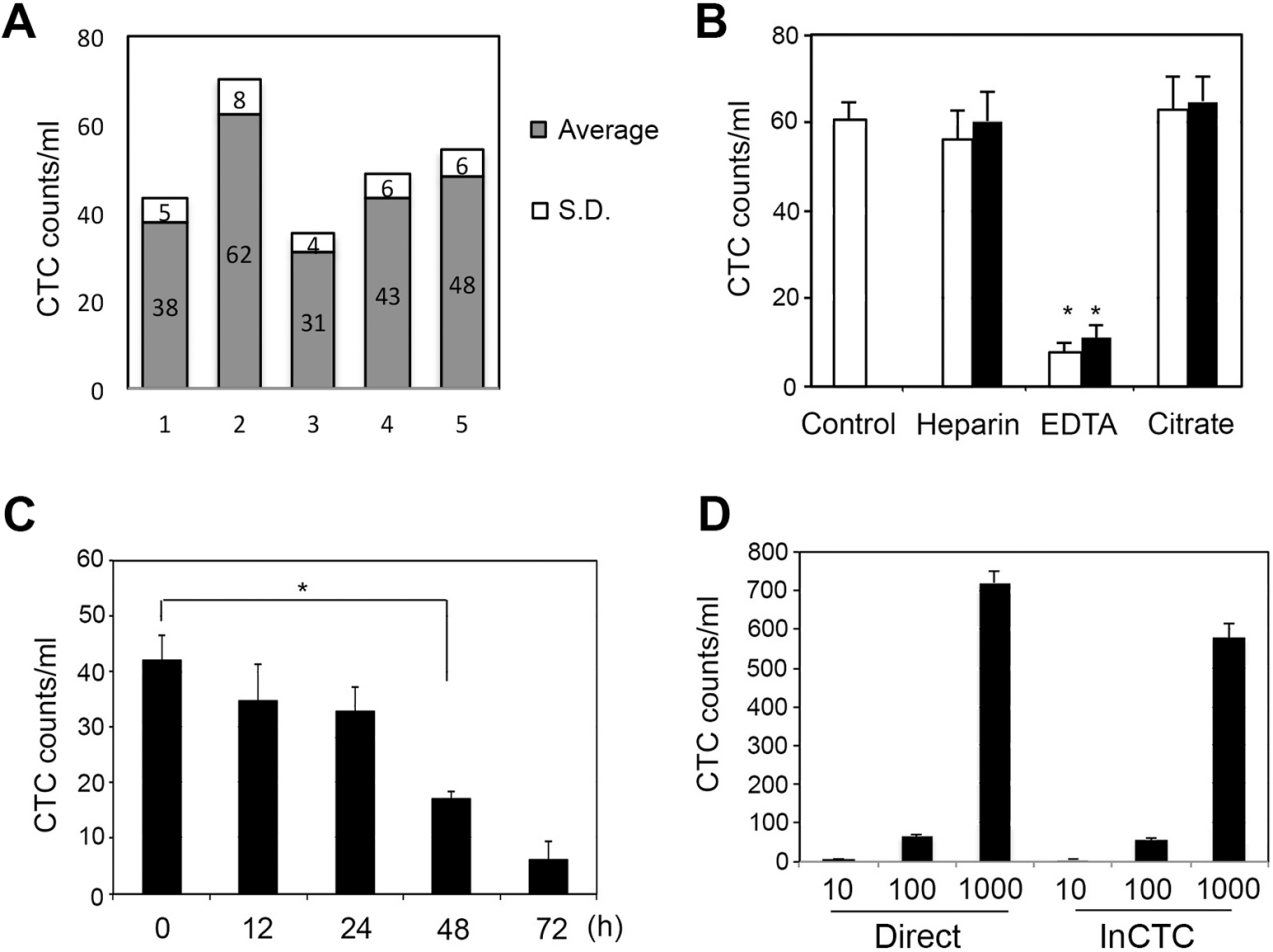
Characterization of InCTC **A.** Variability of InCTC assay. Blood samples were collected from mice bearing H460-GFP tumors. For each mouse, blood samples were divided into 3 equal parts and subjected to InCTC independently. The average and standard deviation (S.D.) of invasive CTCs in each mouse were determined. Data from 5 mice were plotted. **B.** Effect of anticoagulants on InCTC. A total of 100 H460-GFP cells were spiked into 1 ml of normal mouse blood collected using the indicated anticoagulants and subjected to InCTC (black bars). As a control, 100 H460-GFP cells were spiked into 1 ml of PBS containing the same anticoagulants and subjected to InCTC (white bars). The results were derived from 3 independent experiments. Values are mean ± SD. *P < 0.001. **C.** Effect of blood storage on InCTC. Blood samples were collected in heparin from 3 H460-GFP tumor-bearing mice and divided into 5 equal parts after mixing. The samples were stored at 4°C for the indicated times and then subjected to InCTC in triplicates. Values are mean ± SD. *P < 0.01. **D.** Sensitivity of InCTC. A total of 10, 100, or 1, 000 H46-GFP cells was directly loaded onto transwells for invasion assay or were spiked into 1 ml of blood from normal mice and subjected to InCTC. Results were derived from 3 independent experiments. Values are mean ± SD.

We first analyzed the variability of InCTC in detecting CTCs in the blood of 5 mice bearing H460-GFP tumors. For each mouse, blood samples were divided into 3 equal parts and subjected to InCTC independently. The mean number and standard deviation (S.D.) of invasive CTCs in each mouse were determined (Figure 2A). The variation was less than 15%, which suggested that InCTC is consistent in measuring invasive CTCs.

InCTC relies on a cell invasion assay to enumerate CTCs. To evaluate the factors that could affect the results of our method, we first determined the effect of a variety of anticoagulants on blood collection. Heparin and citrate yielded similar InCTC counts, whereas EDTA resulted in substantially lower counts (Figure 2B). We also compared the mononuclear cell-enriching methods of Ficoll-gradient fractioning versus hemolysis on the efficacy of InCTC. For each of 3 H460-GFP tumor-bearing mice, blood samples were collected in heparin. The mononuclear cells were enriched by Ficoll gradient or hemolysis and subjected to cell invasion assay independently. We found that the Ficoll-gradient resulted in higher CTC counts (40 ± 5) compared to hemolysis (21 ± 8).

Next, we explored the effect of blood storage on InCTC. After being stored at 4°C for up to 72 hours, blood from 3 tumor-bearing mice was mixed and divided into 5 equal parts and subjected to InCTC. We observed a time-dependent gradual decrease in CTC counts, and a significant reduction occurred after 48 hours of storage (Figure 2C). Thus, for best results, InCTC may need to be performed within 24 hours after blood collection.

To determine the sensitivity of InCTC in detecting CTCs, a fixed number of H460-GFP cells was either directly applied to the invasion assay or spiked into fresh whole blood collected from normal control mice and subsequently subjected to InCTC. The results showed that 60–70% of the cells were invasive when they were directly loaded into the transwells, whereas 40–60% were invasive when they were spiked into whole blood and subjected to InCTC (Figure 2D). The lower count was largely due to loss of cells during the gradient fractioning steps (data not shown). We were able to detect invasive cells when only 10 H460-GFP cells were spiked into blood (Figure 2D), suggesting that InCTC is highly sensitive.

### Detection of CTCs by InCTC correlates with the presence of metastasis

To demonstrate the general utility, we tested InCTC in xenograft tumor models with different tumor types and genetic backgrounds. Whole blood samples from each of the tumor-bearing mice and control mice from different models were collected and evaluated for the presence of CTCs by InCTC. We did not detect any invasive cytokeratin-positive CTCs in the blood samples from the control athymic nude mice and C57BL/6 mice. In contrast, blood samples from most of tumor-bearing mice had 28–70 invasive CTCs positive for GFP, cytokeratin, or both (Table 1). More interestingly, the few tumor-bearing mice with no detectable CTCs lacked sign of metastasis (Table 1). These results demonstrated that our *in vitro* invasion assay, InCTC, is capable of detecting CTCs in blood of various tumor models and the detection correlated with presence of metastasis.

**Table 1:** InCTC counts in various animal models

Mouse strain	Tumor cell line	CTC counts/ml	Metastasis	Mouse #
Nude mice		0	−	1
		0	−	2
	None	0	−	3
		0	−	4
		0	−	5
		38	+	6
		56	+	7
	NCI-H460	36	+	8
		28	+	9
		32	+	10
		43	+	11
		40	+	12
	NCI-H322	70	+	13
		42	+	14
		40	+	15
		30	+	16
		0	−	17
		48	+	18
	HN31	0	−	19
		0	−	20
		0	−	21
		0	−	22
C57BL/6		0	−	23
		0	−	24
	None	0	−	25
		0	−	26
		0	−	27
		60	+	28
		38	+	29
		57	+	30
		0	−	31
	LLC	0	−	32
		0	−	33
		0	−	34
		0	−	35
		0	−	36

Human lung cancer NCI-H460 and NCI-H322 cells and human head and neck cancer HN31 cells were injected into the flanks of nude mice. Mouse Lewis lung cancer (LLC) cells were injected into the syngeneic C57BL/6 mice. InCTC was performed when 3–4 months after resection of the primary tumor. Metastasis was detected either by *in vivo* imaging of luciferase of the tumor cell or histological examination of lung, liver and head and neck. Each row represents CTC counts from a single mouse.

### InCTC detects more invasive CTCs than EpCAM-based detection method

**Figure 3:**
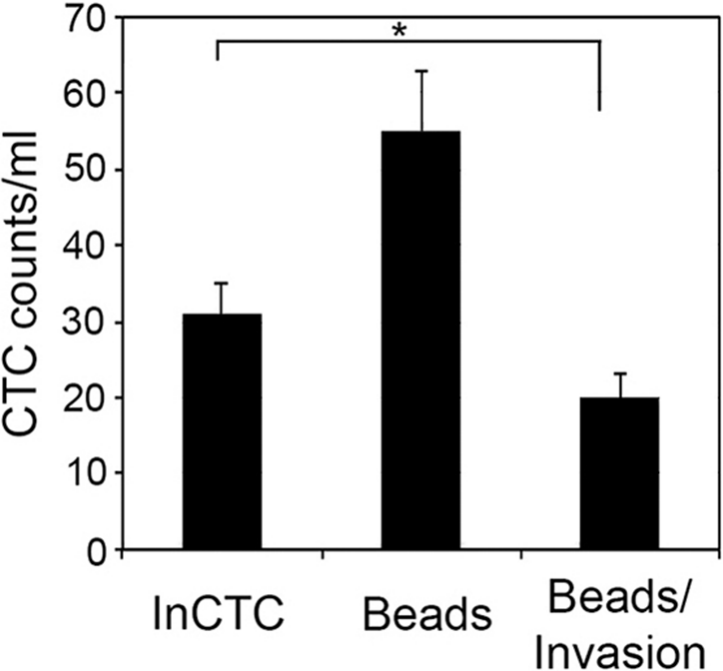
Comparison of InCTC with EpCAM-based immunomagnetic selection For each of 3 H460-GFP tumor-bearing mice, blood samples were divided into 6 equal parts after mixing and subjected to either InCTC or immunomagnetic selection using anti-EpCAM antibody-coupled magnetic beads. The immunomagnetic selected cells were also subjected to transwell invasion assy. Values are mean ± SD. *P < 0.05.

Currently, the most common method of enumerating CTCs is based on immunomagnetic selection of CTCs against the epithelial marker EpCAM. To compare InCTC with this method, blood samples from H460-GFP tumor-bearing mice were divided and subjected to InCTC or to immunomagnetic selection using anti-EpCAM antibody-coupled magnetic beads. The results showed that more CTCs were detected by immunomagnetic selection than by InCTC (Figure 3). However, when the immunomagnetic-selected cells were subjected to invasion assay, less than 50% of the selected cells displayed the ability to invade. More interesting, the number of invasive cells after immunomagnetic selection was lower than the number directly measured by InCTC. Since immunomagnetic selection had no notable effect on the invasion assay (data not shown), these results suggested that although anti-EpCAM antibody-based immunoselection detects more CTCs, it also detects more cells without the invasive phenotype, and that some of the invasive CTCs escape detection by that method.

### Monitoring tumor development using InCTC

**Figure 4:**
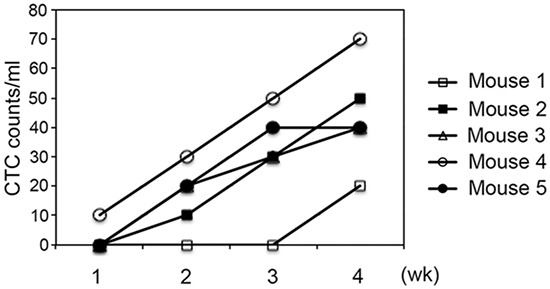
Monitoring tumor growth with InCTC H460-GFP cells were injected into the flank of 5 nude mice. Blood samples (~100 μl) were collected weekly from the tail vein and InCTC was performed. Data for each mouse was plotted.

To demonstrate the utility of InCTC in monitoring tumor development, we injected H460-GFP cells into the flank of 5 nude mice. Blood samples were collected weekly from the tail vein and InCTC was performed. Although there was variation, the CTC count clearly increased with time for each mouse (Figure 4), supporting the idea that InCTC could be used to monitor tumor development.

### Detection of invasive CTCs in patients with lung or esophageal cancer

**Figure 5:**
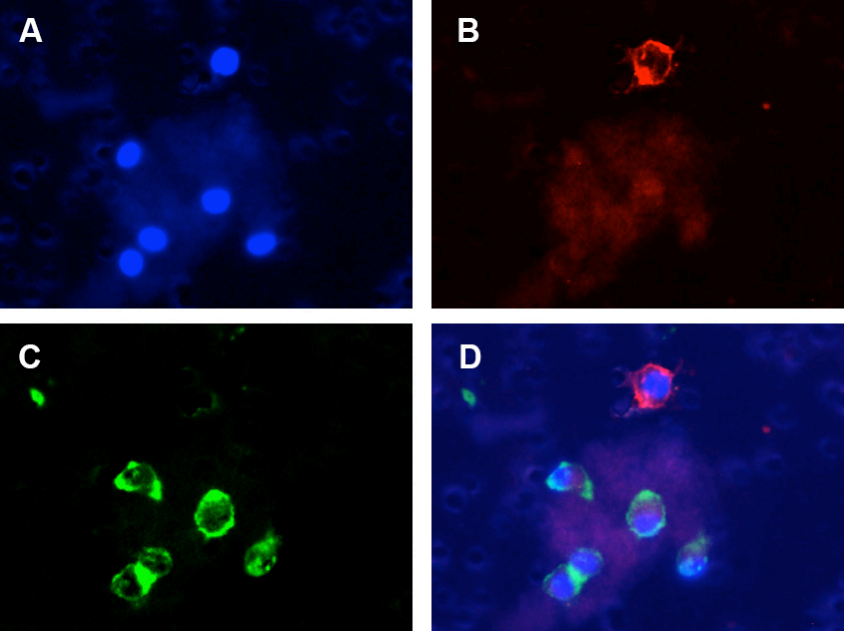
Detection of invasive CTCs in peripheral blood samples of human cancer patients A lung cancer patient blood sample was subjected to InCTC. The invaded cells were stained with DAPI **A.** anti-pan-cytokeratin **B.** and anti-CD45 **C.** and visualized under a fluorescent microscope. **D.** shows a merged image. Representative images are shown.

We have validated the utility of InCTC in xenograft tumor models. To demonstrate that InCTC can be used to detect invasive CTCs in patients with cancer, a small cohort of patients with stage II and III lung or esophageal cancer was selected. Blood samples were collected from these patients before surgery and InCTC was performed. The invaded cells were double-stained with both anti-pan-cytokeratin and anti-CD45 antibodies and DNA was counter stained with DAPI (Figure 5). Cells stained positive for cytokeratin and negative for CD45 were counted. Invasive CTCs were detected in 1 of 4 lung cancer patients and 2 of 2 esophageal cancer patients (Table 2). As a control, no CTCs were detected in the blood samples from healthy donors (data not shown). These results indicate that InCTC is capable of detecting invasive CTCs in human cancer patients.

**Table 2:** Invasive CTC count in cancer patients

Patient number	Cancer type	Staging	CTC counts/5mL
1	Lung adenocarcinoma	IIA	0
2	Lung adenocarcinoma	IIIB	20
3	Lung squamous cell carcinoma	IIIB	0
4	Lung adenocarcinoma	IIA	0
5	Esophageal squamous cell carcinoma	IIIC	2
6	Esophageal squamous cell carcinoma	IIIA	5

Blood samples were collected in heparin tubes and InCTC assays were performed within 24 h. CTC counts were normalized to 5mL.

## DISCUSSION

Detection, enumeration, and subsequent characterization of CTCs at molecular levels not only could provide important insights into disease progression and might allow adaptation of therapeutic strategies, but also shed light on the mechanisms of tumor dissemination and metastasis. A tremendous increase in exploring CTCs in the management of cancer has been seen over the past decade and a number of techniques to refine the sensitivity and range of CTC assays are also in development. However, most, if not all, techniques rely on cell surface marker expression and special equipment. We developed a cell invasion-based assay for CTC detection which we call InCTC. We have demonstrated its feasibility, variability, sensitivity, and utility with mouse xenograft tumor models and in a few cases of human cancer patients. InCTC is a simple technique and needs no special laboratory equipment and thus can be adapted easily in hospitals and research laboratories to promote research on CTCs and metastasis. The major criteria of a good CTC detection and isolation method are high sensitivity and specificity, simplicity of procedure that can be automated or standardized, and low cost. We showed that InCTC is highly sensitive and is capable of detecting as few as 10 CTCs in less than 1 ml of blood. We also showed that InCTC is highly specific, as none of the 10 control mice (Table 1) and an additional 30 C57BL/6 mice (data not shown) produced detectable CTCs. Furthermore, the variability of InCTC was lower than 15%. Because InCTC is based on the invasive property of CTCs and does not rely on the expression of cell markers, it measures only CTCs that are functionally significant and regardless of heterogeneity. This potential is supported by the results that detection of CTCs by InCTC correlated with metastasis in the tumor models (Table 1) and InCTC detected more invasive CTCs than EpCAM-based selection method (Figure 3). Therefore, InCTC could provide us a better opportunity to identify bona fide metastasis-initiating cells and the results generated by InCTC should correlate better with disease outcome and treatment response than current CTC detection methods do.

## MATERIALS AND METHODS

### Generation of tumor-bearing mice

NCI-H460, NCI-H322, and HN31 cells were obtained from the American Type Culture Collection, verified yearly by genomic fingerprinting, and maintained in RPMI-1640 medium for no more than 20 passages. The cells were transfected with a GFP-expressing plasmid (Invitrogen), and stable clones were established and selected for high GFP expression. LLC-GFP cells were a generous gift of Dr. Yi-Ping Li of The University of Texas Medical School in Houston.

All animal experiments were reviewed and approved by the MD Anderson Animal Care and Use Committee. C57BL/6 mice were originally obtained from Jackson Labs and are maintained as part of our breeding colonies. To generate tumors in mice, 1 million tumor cells were implanted into the flank of 4- to 6-week old male athymic nude mice (NCI) for H460-GFP, H322 and HN31 tumors or into the flank of C57BL/6 mice for LLC tumors. When the diameter of a tumor reached to 1 to 1.5 cm, it was surgically removed under anesthesia and euthanized 4 months after surgery. The blood was collected immediately by cardiac puncture for CTC isolation. Lung tissue was also collected and processed for tumor analysis by hematoxylin and eosin staining.

### InCTC assay

To perform the InCTC assay, red blood cells were removed from the blood samples by centrifugation using a Ficoll-Paque premium gradient medium (GE Healthcare) according to the manufacturer’s instruction. The monolayer containing the CTCs and leukocytes was then collected and washed with media without sera. Migration of CTCs was assessed using 8-μm-diameter pore inserts into 24-well plates in a micro-Boyden transwell chamber (Greiner Bio One) with a translucid membrane and coated with Matrigel (1 mg/ml, BD Biosciences). CTCs suspended in medium without serum were seeded onto the upper chamber, and 10% FCS was added to the lower chamber. The transwell chambers were then incubated in a humidified incubator with 5% CO_2_ for 48 hours. After incubation, the inserts were washed with PBS, fixed with ice-cold methanol for 5 minutes, rinsed with PBS, and swabbed with a cotton swab to remove non-migrated cells. The invasive cells were stained with DAPI and an anti-GFP antibody (Santa Cruz Technology) and anti-CK8, 18, 19 antibody (Abcam), or anti-CD45 antibody (R&D Systems). The stained cells were examined under a fluorescence microscope.

### Immunomagnetic selection of CTCs

After the gradient test, the mononuclear cells were incubated with 100 μl of micromagnetic beads coated with anti-EpCAM antibody (Miltenyi-Biotec) and 100 μl of FcR-blocking reagent (Miltenyi-Biotec) for 30 minutes at 4°C and then incubated with 20 μl of magnetic beads coated with anti-CD45 antibody (Miltenyi-Biotec) for 15 minutes at 4°C. The mixture was passed through a magnet-filled column with an AutoMACSPro cell separator system (Miltenyi-Biotec) using the positive selection protocol (POSSELD program) to enrich for EpCAM-positive cells and the negative selection protocol (DEPLETE program) to enrich for CD45-depleted CTCs. The CD45-depleted (i.e., CD45-negative) fraction underwent an additional run through the magnet-filled column using the MACS DEPLETES program to prevent any residual contamination of CD45-positive cells.

### Patient samples

The human study was approved by the Ethics Committee of Zhongshan Hospital of Xiamen University. Patients diagnosed with lung and esophageal cancers were recruited into this study. Written informed consent was obtained from all enrolled patients before blood collection. Peripheral blood samples (3–4 ml, anticoagulated with heparin) were collected before surgery or other treatment and processed within 24 h.

### Statistical analysis

We compared differences between groups via Student’s *t*-test. *P* values < 0.05 were considered to be statistically significant.
